# Measuring the impact of oesophagectomy on physical functioning and physical activity participation: a prospective study

**DOI:** 10.1186/s12885-019-5888-6

**Published:** 2019-07-12

**Authors:** E. M. Guinan, A. E. Bennett, S. L. Doyle, L. O’Neill, J. Gannon, G. Foley, J. A. Elliott, J. O’Sullivan, J. V. Reynolds, J. Hussey

**Affiliations:** 1 School of Medicine, Trinity College Dublin, Dublin, Ireland; 2 Department of Clinical Medicine, School of Medicine, Trinity College Dublin, Dublin, Ireland; 3 School of Biological Sciences, Dublin Institute of Technology, Dublin, Ireland; 4 Discipline of Physiotherapy, School of Medicine, Trinity College Dublin, Dublin, Ireland; 5 Department of Surgery, St. James’ Hospital, Dublin, Ireland; 6 Trinity Translational Medicine Institute, Department of Surgery, Trinity College Dublin, Dublin, Ireland

**Keywords:** Physical functioning, Physical activity, Oesophageal cancer, Survivorship care, Health-related quality of life

## Abstract

**Background:**

Oesophagectomy remains the only curative intervention for oesophageal cancer, with defined nutritional and health-related quality of life (HR-QOL) consequences. It follows therefore that there is a significant risk of decline in physical wellbeing with oesophagectomy however this has been inadequately quantified. This study prospectively examines change in physical functioning and habitual physical activity participation, from pre-surgery through 6-months post-oesophagectomy.

**Methods:**

Patients scheduled for oesophagectomy with curative intent were recruited. Key domains of physical functioning including exercise tolerance (six-minute walk test (6MWT)) and muscle strength (hand-grip strength), and habitual physical activity participation, including sedentary behaviour (accelerometry) were measured pre-surgery (T0) and repeated at 1-month (T1) and 6-months (T2) post-surgery. HR-QOL was measured using the EORTC-QOL C30.

**Results:**

Thirty-six participants were studied (mean age 62.4 (8.8) years, *n* = 26 male, n = 26 transthoracic oesophagectomy). Mean 6MWT distance decreased significantly from T0 to T1 (*p* = 0.006) and returned to T0 levels between T1 and T2 (*p* < 0.001). Percentage time spent sedentary increased throughout recovery (*p* < 0.001) and remained significantly higher at T2 in comparison to T0 (*p* = 0.003). In contrast, percentage time spent engaged in either light or moderate-to-vigorous intensity activity, all reduced significantly (p < 0.001 for both) and remained significantly lower at T2 in comparison to T0 (*p* = 0.009 and *p* = 0.01 respectively). Patients reported deficits in multiple domains of HR-QOL during recovery including global health status (*p* = 0.04), physical functioning (p < 0.001) and role functioning (p < 0.001). Role functioning remained a clinically important 33-points lower than pre-operative values at T2.

**Conclusion:**

Habitual physical activity participation remains significantly impaired at 6-months post-oesophagectomy. Physical activity is a measurable and modifiable target for physical rehabilitation, which is closely aligned with patient-reported deficits in role functioning. Rehabilitation aimed at optimising physical health in oesophageal cancer survivorship is warranted.

## Background

Oesophagectomy is an exemplar model of a complex operation, with a relatively high postoperative risk of major morbidity [1], and defined nutritional and health-related quality of life (HR-QOL) implications [2, 3]. Oesophageal cancer is the eighth most common cancer globally, with an estimated 456,000 new cases in 2012 (3.2% of all cancers) and the sixth most common cause of cancer mortality (4.9% of all cancer deaths) [4]. Approximately 20% of patients diagnosed with oesophageal cancer undergo oesophagectomy with curative intent [5]. This complex procedure involving upper laparotomy usually in combination with thoracotomy and one lung anaesthesia, and is associated with significant postoperative morbidity. Postoperative pulmonary complications (PPCs), which are among the most serious postoperative morbidity, occur in 15–30% of patients post-oesophagectomy and are the primary cause of postoperative mortality, contributing to 45.5–55% of post-oesophagectomy deaths [6]. In the modern era, surgery is preceded by chemotherapy or combination chemoradiotherapy for the majority of patients who present with locally advanced disease [2]; an approach which has contributed to 5-year survival rates of up to 47% [7]. Accordingly, at a time when overall survival is improving, there is a growing emphasis on the nutritional, physical and emotional wellbeing of patients undergoing curative treatment for locally advanced disease [8].

Oesophageal cancer and its treatment, particularly oesophagectomy, leads to significant anatomic and physiologic alterations of the gastrointestinal tract and thus the long-term nutritional implications of curative treatment for oesophageal cancer are well documented [9]. Up to 80% of patients are cachexic at presentation [10], with recent data demonstrating that weight loss, sarcopenia, malabsorption and altered gut hormone function persist into survivorship [11–13]. Notwithstanding the considerable survival advantages of modern multimodal treatment regimens when compared with surgery alone [2, 7], chemotherapy and chemoradiotherapy can adversely impact body composition and muscle strength [14], with emerging evidence linking loss of skeletal muscle mass during neoadjuvant therapy with chemotherapy toxicity and major postoperative complications [15].

It follows therefore that there is a significant risk of decline in physical functioning resulting from both curative treatment for oesophageal cancer and poor nutritional status. Declines in cardiopulmonary fitness, a key determinant of physical functioning [16], ability to engage in activities of daily living [17], and increased risk of postoperative complications [18] are reported with neoadjuvant chemo(radio)therapy [19–21], and associated with higher mortality risk at 1-year post oesophagectomy [21]. Physical inactivity is associated with increased postoperative risk following oesophagectomy [22] and is a defined problem among cancer survivors [23, 24], associated with HR-QOL [25] and, increasingly, survival outcomes [26]. While an acute decline in physical fitness, muscle strength and HR-QOL is described from pre-oesophagectomy to post-operative discharge [17], prospective evaluations characterising the impact of oesophagectomy on physical outcomes, particularly long-term evaluations of physical functioning, are lacking [27]. Subjectively, patients report perceived deficits in physical functioning domains of HR-QOL which persist into survivorship [28, 29]; however, the measured impact of oesophagectomy on physical functioning is inadequately quantified. We have previously described deficits in cardiorespiratory fitness and moderate-to-vigorous intensity physical activity participation in oesophageal cancer survivors at up to two years post-operatively, in comparison to age- and gender-matched controls [30], suggesting that curative treatment exerts a profound and lasting impact on physical status.

With increasing emphasis on survivorship care in oesophageal cancer, there is a recognised need to better understand the physical consequences of oesophageal cancer and its treatment in order to develop tailored rehabilitation programmes involving exercise and diet prescription to attenuate the impact of treatment on physical functioning and optimise HR-QOL in recovery [31]. Cancer survivorship models emphasise that exercise rehabilitation implemented early in the cancer continuum, particularly within the first 6-months postoperatively, is likely to have the greatest impact on HR-QOL [32]. This study therefore seeks to characterise the impact of oesophagectomy on physical functioning and habitual physical activity participation in early postoperative recovery and up to 6-months post-oesophagectomy to inform targets and priorities for exercise rehabilitation during this period.

## Methods

### Study design

Patients with a diagnosis of oesophageal cancer and scheduled for oesophagectomy were identified from the upper gastrointestinal clinic at the Oesophageal and Gastric Centre at St James’s Hospital (SJH), Dublin, Ireland, a high-volume national centre. Ethical approval was obtained from the SJH–Tallaght Hospital Joint Research Ethics Committee. Informed written consent was obtained prior to study commencement. Using a prospective observational design, participants were recruited pre-operatively and measurements were collected pre-surgery (T0), at 1-month post-surgery (T1) and at 6-months post-surgery (T2). Visits were conducted in the Wellcome Trust-HRB Clinical Research Facility at SJH.

### Clinical treatment

All participants were treated according to standardised care pathways involving either multimodal therapy or surgery only. Patients with locally advanced disease received either pre- and postoperative chemotherapy as per the MAGIC regimen [33] or neoadjuvant chemoradiation as per the CROSS protocol [34]. Surgical resection was performed at least 6-weeks post neoadjuvant therapy. The surgical approach involved either transthoracic en-bloc oesophagectomy (2-stage or 3-stage) or transhiatial oesophagectomy following evaluation of patient demographics and comorbidities as previously described [35]. Postoperatively, patients were immediately extubated and admitted to a monitored bed, normally the high dependency unit (HDU). Patients were transferred to the ward on postoperative day (POD)3 or when medically suitable. The institutional enhanced recovery after surgery protocol included the following elements; early enteral feeding via jejunostomy, early mobilisation and airway clearance techniques from POD1, removal of chest drains on POD2 and contrast study for anastomotic integrity on POD4. Postoperative analgesia was managed using thoracic epidural analgesia. The jejunostomy remained in-situ for 4–6 weeks postoperatively and supplemental overnight enteral nutrition was continued on discharge for a planned duration of 4 weeks in all participants [36]. Patients were reviewed at regular intervals postoperatively by the specialist dietetic service.

### Clinical data

Demographic and clinicopathologic data was gathered from medical charts and from the institutional upper gastrointestinal cancer database. Postoperative data included in-hospital mortality, hospital and critical care length of stay (LOS) and postoperative complications.

### Measures of anthropometry

Weight (kg) was recorded using a calibrated Seca scale. Height (cm) was measured barefoot using a Seca stadiometer. Body mass index (BMI) was calculated as weight (kg)/height (m^2^). Mid-arm circumference (MAC) was measured in cm at the halfway point between the olecranon process of the ulna and the acromion process of the scapula. Waist circumference (cm) was measured at the mid-point between the iliac crest and the 12th rib following gentle expiration. MAC and waist circumference were taken in duplicate and averaged for data entry. Bioimpedance analysis was used to determine body composition and was performed using the Seca mBCA 515 (Seca, Hamburg, Germany).

### Measures of physical functioning

Functional exercise performance was measured using the 6-min walk test (6MWT). Participants walked at their fastest pace for 6 min along a 30 m walkway with the aim of achieving the furthest distance possible with standardised verbal encouragement [37]. Isometric hand grip strength (HGS) (kg) was measured using a handheld digital dynamometer (Jamar). Measures were taken in triplicate, bilaterally and the highest measure recorded.

Physical activity was measured using the ActiGraph GT3X+ triaxial accelerometer (Actigraph Pensacola, FL). The accelerometer was worn on the hip, secured with an elastic belt, during waking hours for 7 days following all study visits. Data were analysed using the Actilife software using standardised algorithms to analyse time in physical activity domains and adherence to physical activity guidelines (150 min moderate-to-vigorous intensity physical activity (MVPA)/week, accumulated in bouts ≥10 min [38]). The following cut-points were used to define activity domains: sedentary 0–99 counts per minute (CPM), light 100–2019 CPM, moderate 2020–5998 and vigorous ≥5999 [39]. A valid data was defined as one with 10 h of data and at least four consecutive days were required for analysis.

### Measures of health-related quality of life

QOL was assessed using the European Organisation for Research and Treatment of Cancer (EORTC) Core QOL Questionnaire, the QLQ-C30 (version 3.0). This validated instrument assessed QOL in functional, symptom and global domains. Scores for each question were calculated according to the EORTC QLQ-C30 manual and linearly transformed into a 0–100 scale [40].

### Sample-size considerations

This is an exploratory descriptive study, which considers the impact of oesophagectomy on clinically important outcomes using a patient-centred, multifaceted approach. Using previous literature to estimate an effect size of 0.5 for the effect of treatment for oesophageal cancer on physical status [14] a significance level of 0.05 and a power of 0.8, a sample size of 33 was calculated for repeated measures. This sample estimate is consistent with other publications in this field [17].

### Statistical analyses

SPSS version 22.0 was used for analyses. Variables were tested for normality using the Shapiro-Wilks test. Normally distributed variables were summarised as mean and standard deviation (SD). Non-normally distributed data were summarised as median and interquartile range (IQR). Categorical variables are presented as frequency (percentage).

A mixed between-within subjects analysis of variance (ANOVA) was conducted to assess the change in outcomes across three timepoints in consideration of the impact of treatment regimen (CROSS, MAGIC or surgery only). Data was tested to ensure that the assumptions of homogeneity of variance (Levene’s test for equal variances) and homogeneity of intercorrelations (Box’s M statistic) were not violated. Multivariate statistics were interpreted. Interaction effects were examined and reported where significant. Differences between the groups were presented as partial eta squared *(*η^2^) effect sizes. The strength of the differences was interpreted as small (η^2^ < 0.01), medium (η^2^ = 0.01–0.06) or large (η^2^ = 0.06–0.138). Post-hoc pairwise comparison for comparison of main effects were examined using Bonferroni analysis. A Friedman test was used to examine changes in non-parametric outcomes over the three timepoints. Associations between measures of anthropometry and measures of functional performance were assessed using Pearson Product Moment Correlation Coefficients. Statistical significance was taken at *p* < 0.05.

## Results

**Fig. 1:**
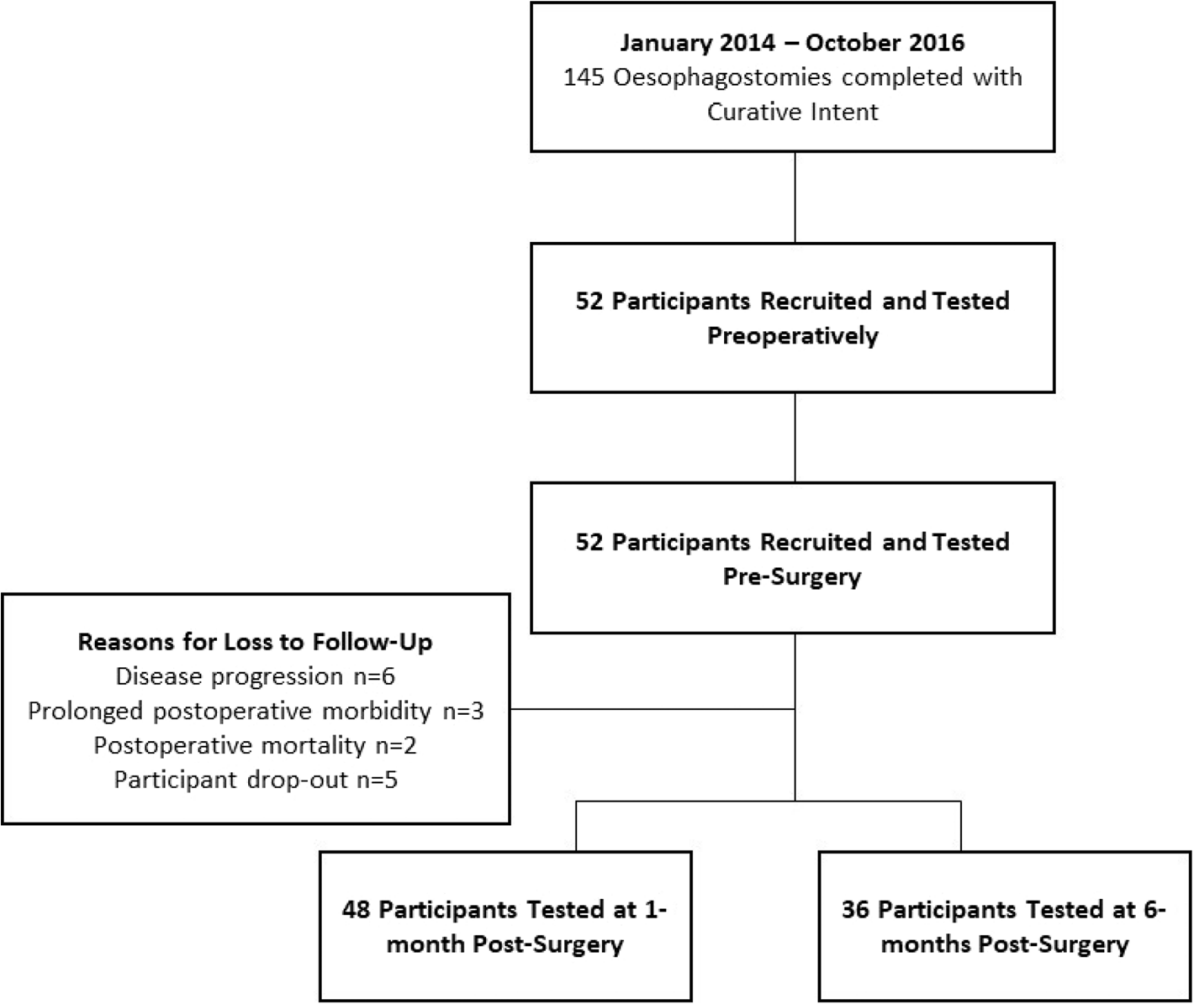
Participant Recruitment and Retention. Flow of participants through the study

Between January 2014 and October 2016, 52 patients were recruited and tested pre-surgery, of whom 43 returned for repeat measures 1-month post-surgery and 36 returned at 6-months post-surgery. The mean time between a pre-surgery assessment and a 1-month post-surgery assessment was 66 (21) days. The mean time between 1-month and 6-months post-surgery assessments was 157 (42) days. Reasons for not completing follow-up measurements were: disease progression (*n* = 6); prolonged postoperative morbidity (*n* = 3); participant death (*n* = 2) and participant drop-out (*n* = 5) (Fig. 1). Demographic characteristics are presented in Table 1. Demographic characteristics of the final sample were comparable to those lost to follow-up.

**Table 1:** Demographic Details

Characteristic	All Participants (*n* = 52)		Participants included in the Final Analysis (*n* = 36)		*p*-value
Age (SD) (years)	62.48 ± 9.03		62.39 (8.82)		0.91
	*n*	%			
Gender					
Male	39	75	26	72	0.73
Female	13	25	10	28	
Smoking Status					
Never	21	40	12	33	N/A
Stopped > 8 weeks preoperative	28	54	22	61	
Stopped < 8 weeks preoperative	1	2	1	3	
Current smoker	2	4	1	3	
Histological Subtype					
Adenocarcinoma	38	73	25	69	0.51
Squamous Cell Carcinoma	14	27	11	31	
Neo-adjuvant therapy protocol					
CROSS	28	54	15	42	N/A
MAGIC	12	23	12	33	
No neoadjuvant therapy	12	23	9	25	
ASA Score					
1	4	8	4	11	N/A
2	35	67	24	67	
3	13	25	8	22	
Surgical Approach					
Transthoracic	40	77	26	72	0.30
Transhiatial	12	23	10	28	
Postoperative Recovery					
Hospital Length of Stay (median (IQR)	13.5 (9)		13.0 (7)		0.86
Critical Care Length of Stay (median (IQR)	3.0 (1.75)		3.0 (1.0)		0.07
Postoperative Complications	31	60	19	53	0.72
In-hospital Postoperative Mortality	1	2	0	0	0.29

Data is displayed as mean (standard deviation (SD)) for normally distributed data and as median (interquartile range (IQR) for non-normally distributed data. Categorical data is presented as frequency (percentage). *P*-value comparing all participants recruited to those included in the final analysis. N/A = chi squared test invalid

### Anthropometry

Body weight, BMI, fat mass, body fat percentage, fat free mass (FFM), skeletal muscle mass, waist circumference and MAC all reduced significantly over the study period (Table 2). Pre-operatively, participants had a mean body weight of 81.9 (16.4) kg and a mean BMI of 27.8 (4.3 kg/m^2^. Male participants had a pre-operative waist circumference of 98.6 (12.3) cm and female participants had a mean waist circumference of 91.38 (9.8) cm. Percentage weight loss from pre-surgery to 1-month (− 6.2 (4.3) %) and to 6-months (− 8.9 (7.4) %) post-surgery was clinically significant. There was no impact of treatment approach on weight (*p* = 0.356, η^2^ = 0.069), BMI (*p* = 0.963, η^2^ = 0.003), fat mass (*p* = 0.0.78, η^2^ = 0.225), body fat percentage (*p* = 0.375, η^2^ = 0.082), skeletal muscle mass (*p* = 0.102, η^2^ = 0.188), waist circumference (*p* = 0.306, η^2^ = 0.102) or MAC (*p* = 0.399, η^2^ = 0.088).

**Table 2:** Change in Measures of Anthropometry Post Oesophagectomy

	Pre-Surgery (T0)	1-month Post-Surgery (T1)	6-Months Post-Surgery (T2)	*P*-value	Multivariate Partial Eta Squared
Anthropometry					
Weight (kg)	81.9 (16.4)	76.9(14.8)*	73.8 (13.2)** ^§^	< 0.001	0.67
Body Mass Index (kg/m^2^)	27.8 (4.3)	26.3 (3.9)*	25.3 (3.7)** ^§^	< 0.001	0.66
Fat Mass (kg)	27.2 (8.7)	25.5 (8.3)*	22.0 (8.6)** ^§^	< 0.001	0.59
Body Fat Percentage (%)	33.6 (7.8)	33.6 (8.2)	30.2 (9.1) ^§^	< 0.001	0.55
Fat Free Mass (kg)	54.5 (9.9)	51.3 (8.8)*	50.7 (11.1) ^§^	< 0.001	0.48
Skeletal Muscle Mass (kg)	27.0 (5.4)	24.6 (4.9)*	24.6 (4.8) ^§^	< 0.001	0.63
Waist Circumference (cm)	97.17 (14.1)	94.5 (11.9)	91.6 (11.2) ^§^	0.002	0.42
Mid-Arm Circumference (cm)	29.8 (3.4)	28.4 (2.9)*	28.9 (2.7)	0.02	0.31

Data is presented as mean (standard deviation) for all variables. P-value for one-way repeated measures ANOVA. *difference between T0 and T1 p < 0.05; ** difference between T1 and T2 *p* < 0.05; ^§^difference between T0 and T2 *p* < 0.05

### Physical functioning

**Fig. 2:**
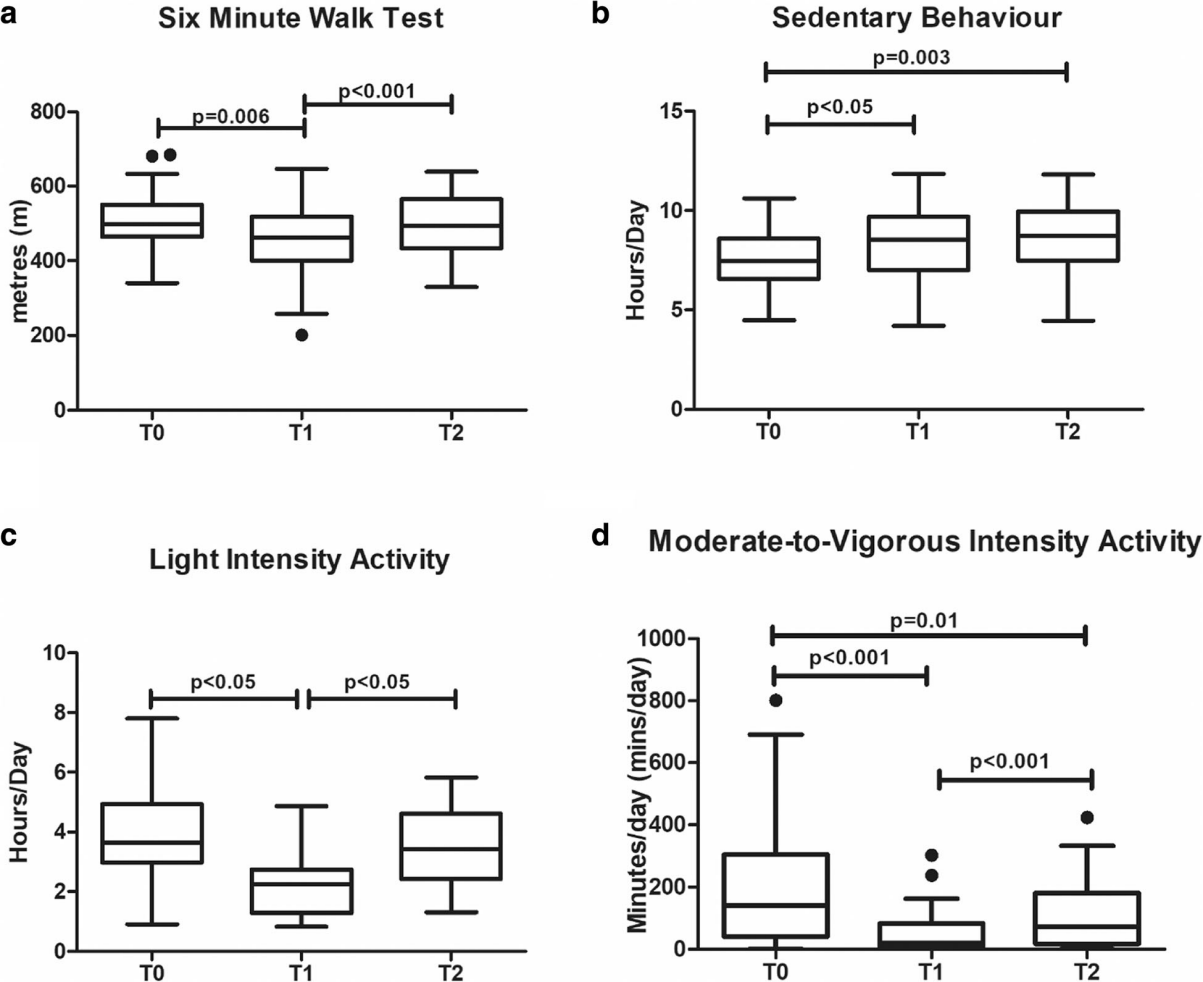
Functional Exercise Tolerance and Physical Activity Levels Pre-Surgery and at 1-Month and 6-Months Post-Surgery. Box-plots depicting distance walked during the six-minute walk test distance (Fig. 1**a**), percentage walking hours spent sedentary (Fig. 1**b**) and in light intensity activity (Fig. 1**c**) and minutes per day spent in moderate-to-vigorous intensity activity (Fig. 1**d**) at each timepoint. All participants experienced significant acute decreases in walk distance and activity levels from pre-surgery to 1-month post-surgery which improved again to 6-months post-surgery, however all domains of physical activity, including sedentary behaviour, remained impaired at 6-months post-surgery in comparison to pre-surgery values

Pre-operatively, male participants walked a mean distance of 513.7 (73.6)m and female participants walked a mean distance of 477.6 (76.0)m during the 6MWT. Significant changes in 6MWT distance were observed over the study period (*p* < 0.001, η^2^ = 0.51) (Table 3). Mean 6MWT distance decreased significantly from pre-surgery (502.6 (76.7)m) to 1-month post-surgery (463.5 (98.4)m) (mean change − 39.1 (95%CI − 68.3 to − 9.9)m, *p* = 0.006), and then increased from 1-month post-surgery to 6-months post-surgery (507.8 (87.8)m) (mean change 44.3 (95%CI 23.0 to 65.5)m, *p* < 0.001) (Fig. 2a). There was no difference between the distance walked pre-surgery and at 6 months post-surgery (*p* = 1.00). There was no impact of treatment approach on 6MWT distance (*p* = 0.639, η^2^ = 0.033). Distance walked during the 6MWT did not correlate with any measure of body composition at any timepoint.

**Table 3:** Functional Performance and Physical Activity Post Oesophagectomy

	Pre-Surgery (T0)	1-month Post-Surgery (T1)	6-Months Post-Surgery (T2)	P-value	Multivariate Partial Eta Squared
Functional Performance					
Six Minute Walk Test Distance (m)	502.6 (76.7)	463.5 (98.4)*	507.8 (87.8)**	< 0.001	0.51
Hand Grip Strength (kg)	35.5 (9.9)	33.9 (9.9)	35.8 (10.9)	0.15	0.15
Physical Activity					
Sedentary behaviour (hours/day)	7.2 (1.6)	8.7 (1.7))*	8.5 (1.7)^§^	0.002	0.46
Light intensity activity (hours/day	4.1 (1.5)	2.3 (0.9)*	3.5 (1.4)**	< 0.001	0.69
Moderate to vigorous intensity activity (minutes/day)	11.5 (31.6)	4.7 (12.9)*	12.5 (24.6)** ^§^	< 0.001	N/A
Adherence to physical activity guidelines	*n* = 11	*n* = 2	*n* = 6	N/A	N/A

Data is presented as mean (standard deviation) for all continuous variables with the exception of moderate to vigorous intensity activity which is presented as median (interquartile range). P-value for one-way repeated measures ANOVA and Friedman’s Test. *mean difference between T0 and T1 p < 0.05; ** difference between T1 and T2 p < 0.05; ^§^difference between T0 and T2 *p* < 0.05

Preoperatively, mean HGS was 38.4 (8.0) kg in males and 23.6 (4.8) kg in females. Mean HGS did not change over time from pre-surgery (35.5 (9.9) kg); neither to 1-month post-surgery (33.9 (9.9) kg) nor to 6-months post-surgery (35.8 (10.9) kg) (*p* = 0.91, η^2^ = 0.15) (Table 3). There was no impact of treatment approach on HGS (*p* = 0.706, η^2^ = 0.025). HGS correlated positively and strongly with skeletal muscle mass at T0 (r = 0.78, *p* < 0.001), T1 (r = 0.73, p < 0.001) and T3 (r = 0.68, p < 0.001).

### Habitual physical activity

In the week before surgery, participants spent 7.2 (1.6) hours/day sedentary, 4.1 (1.5) hours/day engaged in light intensity activity and a median of 11.5 (31.6) minutes/day engaging in moderate-to-vigorous intensity activity. There were significant effects for time for sedentary behaviour (*p* < 0.002, η^2^ = 0.46), light intensity activity (p < 0.001, η^2^ = 0.69), and daily MVPA (p < 0.001) (Table 3). There was no impact of treatment approach on either sedentary behaviour (*p* = 0.766, η^2^ = 0.028) or light intensity activity participation (*p* = 0.694, η^2^ = 0.038).

On post-hoc analysis, sedentary behaviour increased from pre-surgery (7.2 (1.6) hours/day) to 1-month post-surgery (8.7 (1.7) hours per day) (mean change (1.5 (0.39–2.7 h per day), *p* < 0.05) and remained elevated at six-months post-surgery (8.46 (1.7) hours/day) (Fig. 2b). At 6-months post-surgery, sedentary behaviour was significantly higher than pre-operative levels (mean difference 1.3 (0.4–2.2 h/day), *p* = 0.05). Sedentary behaviour did not correlate with skeletal muscle mass at any timepoint.

In contrast, light intensity activity decreased from pre-surgery (4.1 (1.5) hours/day) to 1-month post-surgery (2.3 (0.9) hours per day (mean change − 1.8 (95%CI 2.5 to − 1.1) (p < 0.05) and increased from 1-month post-surgery to six months post-surgery (3.5 (1.4) hours/day (mean change 1.2 (95%CI 0.5 to 1.9) hours/day, p < 0.05) (Fig. 2c). Minutes spent engaging in light intensity activity did not correlate with skeletal muscle mass at any timepoint.

Similarly, daily minutes spent engaging in MVPA, decreased from a median of 11.5 (31.6) minutes/day pre-surgery to 4.7 (12.9) minutes/day at 1-month post-surgery (*p* < 0.001), and increased from 1-month postoperative to 6-months postoperatively (12.5 (24.6) minutes/day (*p* = 0.001) (Fig. 2c). At 6-months post-surgery, daily minutes spent engaging in MVPA was significantly lower than preoperative levels (*p* = 0.01).

### Health-related quality of life

Health-related quality of life scores are detailed in Table 4**.** There were significant changes over time in multiple domains of quality of life on the QLQ-C30 including global health status (*p* = 0.04), physical functioning (p < 0.001), role functioning (p < 0.001), fatigue (p < 0.001), pain (p < 0.001), dyspnoea (p < 0.001), appetite loss (*p* = 0.002) and diarrhoea (*p* = 0.004). Clinically important (> 10-point) change in physical function and role function was reported from pre-surgery to 1-month post-surgery. Role functioning remained impaired (33-points lower) at 6-months post-surgery in comparison with pre-operative values.

**Table 4:** Health Related Quality of Life Post Oesophagectomy

	Pre-Surgery (T0)	1-month Post-Surgery (T1)	6-Months Post-Surgery (T2)	*P*-value
Global Health Status	66.7 (16.7)	58.3 (16.7)*	66.7 (16.7)	0.04
Functional Scales				
Physical Function	93.3 (20.0)	73.3 (40.0)*	86.7 (40.0)**	< 0.001
Role Function	100.0 (33.3)	50.0 (33.3)*	66.7 (33.3)**	< 0.001
Emotional Function	91.7 (33.3)	91.7 (25.0)	91.7 (16.7)	0.337
Cognitive Function	83.3 (33.3)	83.3 (33.3)	100 (16.7)	0.545
Social Function	66.7 (33.3)	66.7 (50.0)	66.7 (50.0)	0.401
Symptom Scales				
Fatigue	22.2 (22.2)	33.3 (22.2)*	33.3 (22.2)**	< 0.001
Nausea/Vomiting	0.0 (16.7)	16.7 (33.3)*	0.0 (16.7)	0.03
Pain	0.0 (16.7)	33.3 (16.7)*	0.0 (33.3)	< 0.001
Dyspnoea	0.0 (0.0)	33.3 (0.0)*	33.3 (33.3)	< 0.001
Insomnia	33.3 (66.7)	33.3 (41.7)	33.3 (33.3)	0.284
Appetite Loss	0.0 (33.3)	33.3 (33.3)*	0.0 (33.3)	0.002
Constipation	0.0 (0.0)	0.0 (33.3)	0.0 (0.0)	0.497
Diarrhoea	0.0 (0.0)	33.3 (33.3)*	0.0 (33.3)	0.004
Financial Difficulties	33.3 (66.7)	33.3 (66.7)	33.3 (66.7)	0.232

Data is presented as median (inter-quartile range) for all variables. P-value for Friedman’s Test

*difference between T0 and T1 p < 0.05; ** difference between T1 and T2 *p* < 0.05

## Discussion

This study provides a novel prospective evaluation of measured and perceived change in physical functioning following oesophagectomy. It demonstrates that habitual physical activity participation and perceived role functioning remain significantly impaired at 6-months post-surgery, despite exercise capacity recovering to preoperative levels. Consistent with previous reports, symptom scores worsened postoperatively [41] and percentage weight loss was clinically significant at 6-months [13]. This significant and clinically relevant pattern of deterioration highlights the challenges of transitioning from active treatment to survivorship post-oesophagectomy and adjusting to the *‘new normal’* after cancer.

The significant short-term and long-term impairments in global HR-QOL, and specifically in physical functioning and symptom burden, that arise following oesophagectomy are well-reported [3, 29, 41–43]. Consistent with the pattern of deterioration in HR-QOL reported by others [41, 42], we observed the largest postoperative impact in the physical functioning domain, specifically in the physical function and role function scales, of the EORTC QOQ-C30. While both scales had improved by 6-months post-surgery, role function remained a clinically relevant 33-points lower in comparison with pre-operative values. Clinically important deteriorations in physical functioning are reported with all curative interventions for oesophageal cancer, including neoadjuvant therapy, minimally invasive surgery and open surgical resection [29]. Importantly however, despite improvements in the majority of HR-QOL domains over time, patient-perceived physical functioning remains impaired at 1-year [41], 2-years [44] and up to 3-years post oesophagectomy [43]. Uniquely, we demonstrate that this profound patient-perceived deterioration in physical health is matched by clinically relevant changes in habitual activity participation.

Functional decline associated with cancer treatment is typically examined using validated, objective measures of physical functioning [16]. Using this approach, we observed an acute deterioration in physical activity participation, as measured by accelerometry, at 1-month post-oesophagectomy, with physical activity levels and sedentary behaviour remaining impaired at 6-months. Consistent with this, we have previously reported that moderate-to-vigorous intensity activity participation is significantly lower at 2-years post-oesophagectomy in comparison with age- and gender-matched controls [30]. Importantly, accelerometry captures habitual activity participation during waking hours and therefore, engagement in activities of daily living, a construct that is well-aligned with the role functioning domain of the EORTC QLQ-C30 [45], which was were perceived by participants to remain considerably impaired in survivorship. Role functioning considers an individual’s ability to engage in activities that are typical for their age and social setting [45]. In patients with cancer, physical activity levels are known to reduce at cancer diagnosis and rarely return to baseline levels following treatment completion [24]. Compared to those who are inactive or sub-optimally active, cancer survivors who exercise to recommended levels consistently report higher HR-QOL scores, particularly in physical and role functioning domains [25], hypothesised to be driven by the positive effects of physical fitness on mental wellbeing and social engagement [46], making physical activity an important and influential target in survivorship care.

Cardiopulmonary fitness, the primary measure of physical functioning [16], is impaired by the iterative attritional impact of multimodal treatment regimens in oesophageal cancer [27]. We observed a large, clinically important reduction (− 39.10 (95%CI − 68.28 to − 9.92) m) in 6MWT distance at 1-month post-oesophagectomy, consistent with the decline previously reported in a Japanese cohort from pre-surgery (563.3 (73.2) m) to pre-hospital discharge (485.3 (85.6) m) [17]. In colorectal resection, 6MWT distance at 4-weeks post-surgery is discriminative of older age, poorer physical status, open resection and occurrence of postoperative complications, and therefore is a valuable indicator of early physical recovery [47]. While the 6MWT provides a valid measure of functional exercise status in patients with cancer [48], reliability and reproducibility data is lacking [16], and walking distances correlate poorly with cardiopulmonary fitness in comparison with incremental walking protocols [49]. In contrast to our observation that 6MWT distance returned to preoperative values at 6-months postoperatively, suggesting an element of natural recovery in this cohort, a study in a Japanese cohort, utilising the highly sensitive cardiopulmonary exercise test, reported a reduction in exercise capacity from 1186.6 (300.30) ml/min pre-oesophagectomy to 916.1 (238.6) ml/min 3-months postoperatively (*p* < 0.0001) following open resection [50]. Furthermore, we previously reported significantly lower walking distance in disease-free patients up to 2-years post-oesophagectomy (558.33 (146.43)m) in comparison with age- and gender-matched controls (773.48 (114.00)m) using a progressive, incremental walking protocol [30]. Therefore, it is likely that cardiopulmonary fitness remains impaired in oesophageal cancer survivorship; however further prospective evaluations using sensitive measures of fitness are required.

The nutritional challenge of recovery post-oesophagectomy is well-documented. Consistent with previous reports, in this cohort weight loss remained significant at 6-months [13] and symptom burden was considerable in early post-operative recovery. While HGS, a reliable indicator of whole-body muscle strength and nutritional status [51], remained stable over the study period, we have previously reported significant deficits in grip strength with loss of lean body mass during neo-adjuvant therapy [14]. Furthermore, sarcopenia remains prevalent in survivorship with 35% of patients sarcopenic at 1-year post-oesophagectomy [13]. This complex interplay between nutritional insufficiency and physical deterioration makes survivorship rehabilitation particularly challenging. We recently designed, implemented and evaluated the Rehabilitation Strategies Following Oesophagogastric Cancer (ReStOre) programme, a rehabilitation programme for oesophagogastric survivorship comprising exercise training, individualised dietary counselling and multidisciplinary education, with a strong focus on self-management [52–54], leading to clinically important improvements in cardiopulmonary fitness [52], inflammatory status [53], and multiple domains of HR-QOL [54]. The ReStOre programme, the first exemplar in oesophagogastric cancer rehabilitation, included participants up to 5-years post-surgery, however rehabilitative measures implemented earlier in survivorship, particularly within the first 6-months, are likely to have greatest effect [32] and address the issues identified by this work. Consistent with established clinical rehabilitation models, cancer rehabilitation commencing from diagnosis and continuing through the treatment trajectory, may have a key role in attenuating the impact of multiple attritional oncologic treatments, optimising patient condition for surgical intervention and supporting patients through recovery and into survivorship [8, 31].

This work has some limitations which are acknowledged. Firstly, participant retention was challenging with 69% of those initially recruited (*n* = 52) available for evaluation at 6-months (*n* = 36). This is an inevitable challenge of prospective data collection in a cohort undergoing complex surgical and medical interventions. Reasons for attrition are reported and were largely attributed to disease progression and protracted postoperative morbidity. Importantly, those included in the final analyses had comparable baseline characteristics to those lost to follow-up. Nonetheless, the final study cohort represent those who are recovering relatively well at 6 months post-surgery and therefore generalisability is limited. The sample size is comparable to other published work in this field [17, 30, 50]. The use of objective measures of physical functioning is a considerable strength of this work. By employing these methods, multiple measurable and modifiable targets for physical rehabilitation were identified which were well-aligned with patient-reported survivorship issues.

## Conclusions

These results add to the growing evidence that improvements in oncological outcomes in oesophageal cancer have led to a newly emergent cohort of cancer survivors with considerable physical and nutritional concerns. Importantly, results identify deficits in both perceived role functioning and measured activity participation in recovery, suggesting that patients experience considerable challenges adjusting to the ‘*new normal’* in survivorship. Multidisciplinary rehabilitation with a strong focus on self-management and overcoming barriers to habitual activity participation is warranted.

## Data Availability

The datasets used and/or analysed during the current study are available from the corresponding author on reasonable request.

## Abbreviations

6MWT: 6-min walk test
BMI: Body mass index
EORTC: European Organisation for Research and Treatment of Cancer
FFM: Fat free mass
HDU: High dependency unit
HR-QOL: Health-related quality of life
IQR: Interquartile range
LOS: Length of stay
MAC: Mid-arm circumference
MWPA: Moderate-to-vigorous intensity physical activity
POD: Postoperative day
SD: Standard deviation

## References

[1] Low DE, Alderson D, Cecconello I, Chang AC, Darling GE, D'Journo XB, Griffin SM, Holscher AH, Hofstetter WL, Jobe BA (2015). International consensus on standardization of data collection for complications associated with Esophagectomy: Esophagectomy complications consensus group (ECCG). Ann Surg.

[2] Reynolds JV, Preston SR, O'Neill B, Baeksgaard L, Griffin SM, Mariette C, Cuffe S, Cunningham M, Crosby T, Parker I (2017). ICORG 10-14: NEOadjuvant trial in adenocarcinoma of the oEsophagus and oesophagoGastric junction international study (neo-AEGIS). BMC Cancer.

[3] Donohoe CL, McGillycuddy E, Reynolds JV (2011). Long-term health-related quality of life for disease-free esophageal cancer patients. World J Surg.

[4] Wong MCS, Hamilton W, Whiteman DC, Jiang JY, Qiao Y, Fung FDH, Wang HHX, Chiu PWY, Ng EKW, Wu JCY (2018). Global incidence and mortality of oesophageal cancer and their correlation with socioeconomic indicators temporal patterns and trends in 41 countries. Sci Rep.

[5] Allum WH, Blazeby JM, Griffin SM, Cunningham D, Jankowski JA, Wong R (2011). Guidelines for the management of oesophageal and gastric cancer. Gut.

[6] Feeney C, Hussey J, Carey M, Reynolds JV (2010). Assessment of physical fitness for esophageal surgery, and targeting interventions to optimize outcomes. Diseases of the esophagus : official journal of the International Society for Diseases of the Esophagus / ISDE.

[7] Shapiro J, van Lanschot JJ, Hulshof MC, van Hagen P, van Berge Henegouwen MI, Wijnhoven BP, van Laarhoven HW, Nieuwenhuijzen GA, Hospers GA, Bonenkamp JJ (2015). Neoadjuvant chemoradiotherapy plus surgery versus surgery alone for oesophageal or junctional cancer (CROSS): long-term results of a randomised controlled trial. Lancet Oncol.

[8] Guinan EM, Dowds J, Donohoe C, Reynolds JV, Hussey J (2017). The physiotherapist and the esophageal cancer patient: from prehabilitation to rehabilitation. Diseases of the esophagus : official journal of the International Society for Diseases of the Esophagus / ISDE.

[9] Ryan AM, Healy LA, Power DG, Rowley SP, Reynolds JV (2007). Short-term nutritional implications of total gastrectomy for malignancy, and the impact of parenteral nutritional support. Clin Nutr.

[10] Donohoe CL, Ryan AM, Reynolds JV: Cancer cachexia: mechanisms and clinical implications. Gastroenterol Res Pract 2011, 2011:601434. 10.1155/2011/601434 PMC3132494 21760776

[11] Elliott JA, Docherty NG, Eckhardt HG, Doyle SL, Guinan EM, Ravi N, Reynolds JV, le Roux CW (2016). Weight loss, satiety, and the postprandial gut hormone response after Esophagectomy: a prospective study. Ann Surg.

[12] Heneghan HM, Zaborowski A, Fanning M, McHugh A, Doyle S, Moore J, Ravi N, Reynolds JV (2015). Prospective study of malabsorption and malnutrition after esophageal and gastric Cancer surgery. Ann Surg.

[13] Elliott JA, Doyle SL, Murphy CF, King S, Guinan EM, Beddy P, Ravi N, Reynolds JV (2017). Sarcopenia: prevalence, and impact on operative and oncologic outcomes in the multimodal Management of Locally Advanced Esophageal Cancer. Ann Surg.

[14] Guinan EM, Doyle SL, Bennett AE, O'Neill L, Gannon J, Elliott JA, O'Sullivan J, Reynolds JV, Hussey J (2018). Sarcopenia during neoadjuvant therapy for oesophageal cancer: characterising the impact on muscle strength and physical performance. Supportive care in cancer : official journal of the Multinational Association of Supportive Care in Cancer.

[15] Prado CM, Lieffers JR, McCargar LJ, Reiman T, Sawyer MB, Martin L, Baracos VE (2008). Prevalence and clinical implications of sarcopenic obesity in patients with solid tumours of the respiratory and gastrointestinal tracts: a population-based study. Lancet Oncol.

[16] Granger CL, McDonald CF, Parry SM, Oliveira CC, Denehy L (2013). Functional capacity, physical activity and muscle strength assessment of individuals with non-small cell lung cancer: a systematic review of instruments and their measurement properties. BMC Cancer.

[17] Tatematsu N, Hasegawa S, Tanaka E, Sakai Y, Tsuboyama T (2013). Impact of oesophagectomy on physical fitness and health-related quality of life in patients with oesophageal cancer. Eur J Cancer Care (Engl).

[18] Whibley J, Peters CJ, Halliday LJ, Chaudry AM, Allum WH (2018). Poor performance in incremental shuttle walk and cardiopulmonary exercise testing predicts poor overall survival for patients undergoing esophago-gastric resection. European journal of surgical oncology : the journal of the European Society of Surgical Oncology and the British Association of Surgical Oncology.

[19] Lund M, Alexandersson von Döbeln G, Nilsson M, Winter R, Lundell L, Tsai JA, Kalman S (2015). Effects on heart function of neoadjuvant chemotherapy and chemoradiotherapy in patients with cancer in the esophagus or gastroesophageal junction – a prospective cohort pilot study within a randomized clinical trial. Radiat Oncol.

[20] von Dobeln GA, Nilsson M, Adell G, Johnsen G, Hatlevoll I, Tsai J, Lundell L, Lund M, Lind P (2016). Pulmonary function and cardiac stress test after multimodality treatment of esophageal cancer. Pract Radiat Oncol.

[21] Jack S, West MA, Raw D, Marwood S, Ambler G, Cope TM, Shrotri M, Sturgess RP, Calverley PM, Ottensmeier CH (2014). The effect of neoadjuvant chemotherapy on physical fitness and survival in patients undergoing oesophagogastric cancer surgery. European journal of surgical oncology : the journal of the European Society of Surgical Oncology and the British Association of Surgical Oncology.

[22] Feeney C, Reynolds JV, Hussey J (2011). Preoperative physical activity levels and postoperative pulmonary complications post-esophagectomy. Diseases of the esophagus : official journal of the International Society for Diseases of the Esophagus / ISDE.

[23] Guinan EM, Connolly EM, Kennedy MJ, Hussey J (2013). The presentation of metabolic dysfunction and the relationship with energy output in breast cancer survivors: a cross-sectional study. Nutr J.

[24] Broderick JM, Hussey J, Kennedy MJ, O'Donnell DM (2014). Testing the 'teachable moment' premise: does physical activity increase in the early survivorship phase?. Supportive care in cancer : official journal of the Multinational Association of Supportive Care in Cancer.

[25] Husson O, Mols F, Ezendam NP, Schep G (2015). Van de poll-Franse LV: health-related quality of life is associated with physical activity levels among colorectal cancer survivors: a longitudinal, 3-year study of the PROFILES registry. J Cancer Surviv.

[26] Ballard-Barbash R, Friedenreich CM, Courneya KS, Siddiqi SM, McTiernan A, Alfano CM (2012). Physical activity, biomarkers, and disease outcomes in cancer survivors: a systematic review. J Natl Cancer Inst.

[27] ON L, Moran J, Guinan EM, Reynolds JV, Hussey J (2018). Physical decline and its implications in the management of oesophageal and gastric cancer: a systematic review. J Cancer Surviv.

[28] Daster S, Soysal SD, Stoll L, Peterli R, von Flue M, Ackermann C (2014). Long-term quality of life after Ivor Lewis esophagectomy for esophageal cancer. World J Surg.

[29] Scarpa M, Valente S, Alfieri R, Cagol M, Diamantis G, Ancona E, Castoro C (2011). Systematic review of health-related quality of life after esophagectomy for esophageal cancer. World journal of gastroenterology : WJG.

[30] Gannon JA, Guinan EM, Doyle SL, Beddy P, Reynolds JV, Hussey J (2017). Reduced fitness and physical functioning are long-term sequelae after curative treatment for esophageal cancer: a matched control study. Diseases of the esophagus : official journal of the International Society for Diseases of the Esophagus / ISDE.

[31] O'Neill L, Gannon J, Guinan EM, Reynolds JV, Hussey J (2017). Multidisciplinary rehabiltiation across the esophageal cancer journey. J Thoracic Dis.

[32] Courneya KS, Friedenreich CM (2001). Framework PEACE: an organizational model for examining physical exercise across the cancer experience. Ann Behav Med.

[33] Cunningham D, Allum WH, Stenning SP, Thompson JN, Van de Velde CJ, Nicolson M, Scarffe JH, Lofts FJ, Falk SJ, Iveson TJ (2006). Perioperative chemotherapy versus surgery alone for resectable gastroesophageal cancer. N Engl J Med.

[34] van Hagen P, Hulshof MC, van Lanschot JJ, Steyerberg EW, van Berge Henegouwen MI, Wijnhoven BP, Richel DJ, Nieuwenhuijzen GA, Hospers GA, Bonenkamp JJ (2012). Preoperative chemoradiotherapy for esophageal or junctional cancer. N Engl J Med.

[35] Donohoe CL, O'Farrell NJ, Ravi N, Reynolds JV (2012). Evidence-based selective application of transhiatal esophagectomy in a high-volume esophageal center. World J Surg.

[36] Donohoe CL, Healy LA, Fanning M, Doyle SL, Hugh AM, Moore J, Ravi N, Reynolds JV (2017). Impact of supplemental home enteral feeding postesophagectomy on nutrition, body composition, quality of life, and patient satisfaction. Diseases of the esophagus : official journal of the International Society for Diseases of the Esophagus / ISDE.

[37] ATS statement: guidelines for the six-minute walk test. American journal of respiratory and critical care medicine 2002, 166(1):111–117. 10.1164/ajrccm.166.1.at1102 12091180

[38] Schmitz KH, Courneya KS, Matthews C, Demark-Wahnefried W, Galvao DA, Pinto BM, Irwin ML, Wolin KY, Segal RJ, Lucia A (2010). American College of Sports Medicine roundtable on exercise guidelines for cancer survivors. Med Sci Sports Exerc.

[39] Troiano RP, Berrigan D, Dodd KW, Masse LC, Tilert T, McDowell M (2008). Physical activity in the United States measured by accelerometer. Med Sci Sports Exerc.

[40] Fayers PAN, Bjordal K, Groenvold M, Curran D, Bottomley A. The EORTC QLQ-C30 scoring manual. In*.*, 3rd edition edn. Brussels: European organisation for research and treatment of. Cancer. 2001.

[41] Reynolds JV, McLaughlin R, Moore J, Rowley S, Ravi N, Byrne PJ (2006). Prospective evaluation of quality of life in patients with localized oesophageal cancer treated by multimodality therapy or surgery alone. Br J Surg.

[42] van Meerten E, van der Gaast A, Looman CW, Tilanus HW, Muller K, Essink-Bot ML (2008). Quality of life during neoadjuvant treatment and after surgery for resectable esophageal carcinoma. Int J Radiat Oncol Biol Phys.

[43] Lagergren P, Avery KN, Hughes R, Barham CP, Alderson D, Falk SJ, Blazeby JM (2007). Health-related quality of life among patients cured by surgery for esophageal cancer. Cancer.

[44] Schneider L, Hartwig W, Aulmann S, Lenzen C, Strobel O, Fritz S, Hackert T, Keller M, Buchler MW, Werner J (2010). Quality of life after emergency vs. elective esophagectomy with cervical reconstruction. *Scandinavian journal of surgery : SJS : official organ for the Finnish Surgical Society and the*. Scandinavian Surgical Society.

[45] Gamper EM, Petersen MA, Aaronson N, Costantini A, Giesinger JM, Holzner B, Kemmler G, Oberguggenberger A, Singer S, Young T (2016). Development of an item bank for the EORTC role functioning computer adaptive test (EORTC RF-CAT). Health Qual Life Outcomes.

[46] Dimeo FC (2001). Effects of exercise on cancer-related fatigue. Cancer.

[47] Pecorelli N, Fiore JF, Gillis C, Awasthi R, Mappin-Kasirer B, Niculiseanu P, Fried GM, Carli F, Feldman LS (2016). The six-minute walk test as a measure of postoperative recovery after colorectal resection: further examination of its measurement properties. Surg Endosc.

[48] Schmidt K, Vogt L, Thiel C, Jager E, Banzer W (2013). Validity of the six-minute walk test in cancer patients. Int J Sports Med.

[49] Moran J, Wilson F, Guinan E, McCormick P, Hussey J, Moriarty J (2016). The preoperative use of field tests of exercise tolerance to predict postoperative outcome in intra-abdominal surgery: a systematic review. J Clin Anesth.

[50] Taguchi S, Osugi H, Higashino M, Tokuhara T, Takada N, Takemura M, Lee S, Kinoshita H (2003). Comparison of three-field esophagectomy for esophageal cancer incorporating open or thoracoscopic thoracotomy. Surg Endosc.

[51] Cruz-Jentoft AJ, Baeyens JP, Bauer JM, Boirie Y, Cederholm T, Landi F, Martin FC, Michel JP, Rolland Y, Schneider SM (2010). Sarcopenia: European consensus on definition and diagnosis: report of the European working group on sarcopenia in older people. Age Ageing.

[52] O'Neill L, Guinan E, Doyle SL, Elliott JA, O'Sullivan J, Reynolds JV, Hussey J (2017). Rehabilitation strategies following esophageal cancer (the ReStOre trial): a feasibility study. Dis Esophagus.

[53] Guinan EM, Doyle SL, O'Neill L, Dunne MR, Foley EK, O'Sullivan J, Reynolds JV, Hussey J (2016). Effects of a multimodal rehabilitation programme on inflammation and oxidative stress in oesophageal cancer survivors: the ReStOre feasibility study. Supportive care in cancer : official journal of the Multinational Association of Supportive Care in Cancer.

[54] Bennett AM, ON L, Connolly D, Guinan EM, Boland L, Doyle SL, O'Sullivan J, Reynolds JV, Hussey J (2018). Patient experiences of a physiotherapy-led multidisciplinary rehabilitative intervention after successful treatment for oesophago-gastric cancer. Supportive care in cancer : official journal of the Multinational Association of Supportive Care in Cancer.

